# Synthesis, Biological Evaluation, and Pharmacokinetic Study of Novel Liguzinediol Prodrugs

**DOI:** 10.3390/molecules18044561

**Published:** 2013-04-18

**Authors:** Zheng Liu, Wei Li, Hong-Mei Wen, Hui-Min Bian, Jing Zhang, Lei Chen, Long Chen, Kun-Di Yang

**Affiliations:** School of Pharmacy, Nanjing University of Chinese Medicine, 138 Xianlin Road, Nanjing 210046, Jiangsu, China

**Keywords:** liguzinediol, liguzinediol prodrugs, synthesis, positive inotropic effect, pharmacokinetics

## Abstract

Liguzinediol (LZDO) ester prodrugs **3**–**5** were synthesized and evaluated *in vitro* and *in vivo* for their potential use in prolonging the half-life of the parent drug LZDO (**1a**)* in vivo*. Prodrugs **3**–**5** were found to display a potent positive inotropic effect on the myocardium, without the risk of arrhythmia. Prodrugs **3**–**5** rapidly underwent enzymatic hydrolysis to release the parent compound LZDO in 1–3 h in rat liver microsomes and rat plasma. The half-life of the parent compound was prolonged after intragastric administration of prodrug **3**, which was found to be a superior prodrug candidate for increasing myocardial contractility.

## 1. Introduction

Cardiotonic agents exert their therapeutic activity by inducing positive inotropic effects on the myocardium. They are either cAMP-dependent or cAMP-independent, based upon their mechanism of action, but this mechanism of action is inherently responsible for the heart side effects observed in patients undergoing a long-term treatment. The most common effects associated with cardiotonic agents therapy are high mortality [1,2,3,4,5,6], myocardial ischemia aggravation [7,8], arrhythmia, hypotension [9], and increased heart rate [10].

**Figure 1:**
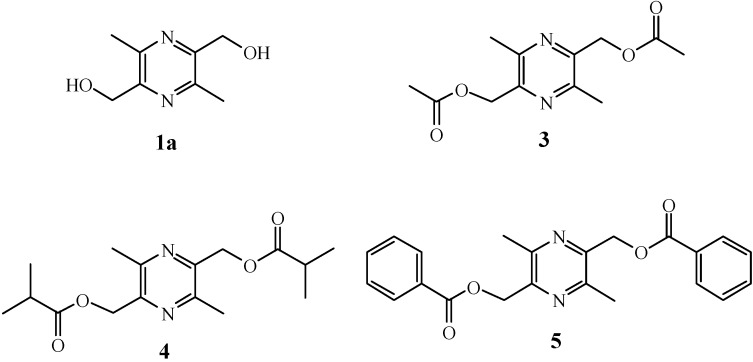
Chemical structures of LZDO (**1a**) and LZDO prodrugs **3**–**5**.

Liguzinediol (LZDO, **1a**, Figure 1) is a *para*-dihydroxy derivative of ligustrazine (**1**), which was isolated from the traditional Chinese medicine herb *Chuanxiong* (*Ligusticum wallichii* Franch). Ligustrazine (**1**) has shown cardiac effects in animal experiments and suppressed l-type Ca^2+^ channel current in rat ventricular myocytes [11,12,13,14,15,16]. We recently found that LZDO (which is patented [17]) exerted positive inotropic effects without the risk of arrhythmia, and its positive inotropic effect in isolated rat hearts was mediated through an elevation of sarcoplasmic reticulum (SR) Ca^2+^ transient, which may act on SR Ca^2+^ ATPase. LZDO (**1a**) thus has a unique biological mechanism that may prove effective in clinically treating heart failure [18]. Moreover, a pharmacokinetic study [19] of LZDO (**1a**) showed that its half-life was about 2 h and its clearance was approximately 0.9 L/h·kg after intragastric administration, suggesting that elimination or biotransformation of LZDO was relatively quick in rats, and that LZDO may be mainly indicated for the treatment of acute heart failure and acute paroxysmal phase of chronical heart failure. It is necessary to modify molecular structure of LZDO and study pharmacodynamics of the derivatives, in order to improve treatment compliance among heart failure patients, satisfy different drug delivery and dosage form clinically. Prodrug formation has been considered as a useful approach to prolong the half-life of the parent drugs and effective drug duration *in vivo*. The general rationale behind the prodrug strategy is to introduce lipophilicity and mask hydrogen bonding groups of an active compound by the addition of another moiety, most commonly an ester. Bioreversible esters have received considerable attention because of the presence of enzymes in the living system capable of hydrolyzing them. In both drug discovery and development, prodrugs have become an established tool for improving physicochemical, biopharmaceutical, or pharmacokinetic properties of pharmacologically potent compounds, thereby increasing the developability and usefulness of a potential drug [20,21].

In the present study, prodrugs **3**–**5** were prepared and evaluated for potential drug application. The inotropic effects on the myocardium in normal isolated rat hearts, metabolic stability, formation of drug from prodrug in rat liver microsomes and rat plasma, and pharmacokinetics of the parent compound in rat plasma after intragastric administration of prodrug **3** have been studied in comparison with the parent drug LZDO (**1a**).

## 2. Results and Discussion

LZDO ester prodrugs **3**–**5** were synthesized with the aim of obtaining enzymatically labile drugs, prolonging the half-life of the parent drug **1a** *in vivo*. The prodrugs were evaluated *in vitro* and *in vivo* for their potential use as prodrugs. 

### 2.1. Chemistry

**Scheme 1:**
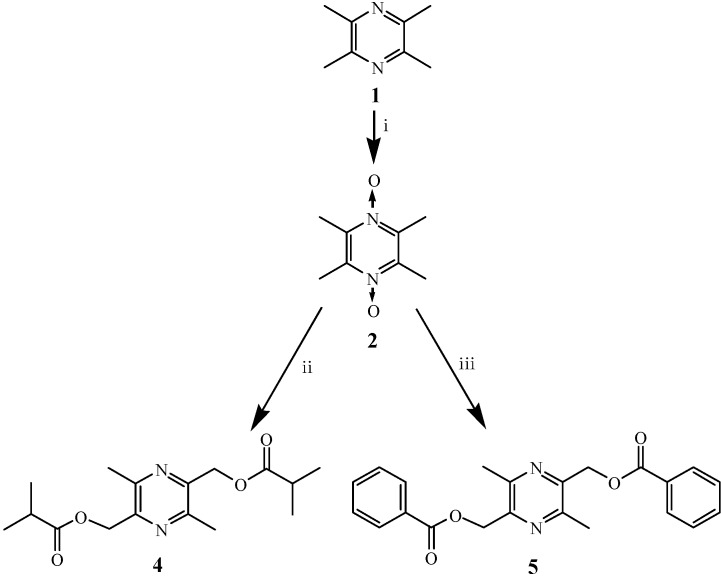
Synthesis of LZDO prodrugs **4**, **5**.

The key intermediate *N*,*N'*-dioxotetramethylpyrazine (**2**), prodrug **3** (34.1% yield), and LZDO (**1a**) were prepared as previously reported [22]. The novel LZDO ester prodrugs **4**, **5** were synthesized in 18.5% and 36.8% yield, respectively, in a straightforward manner starting from the key intermediate **2**, by treatment of the latter with acetic anhydride, isobutyric anhydride, and benzoyl chloride, respectively, as illustrated in Scheme 1. The structures of all prodrug compounds were established by ^1^H-NMR, ^13^C-NMR, IR, and mass spectrometry, and their purity in excess of 98.0% was confirmed by HPLC analysis.

*Reagents and conditions*: (**i**) 30% H_2_O_2_, glacial acetic acid, 98 °C, 12 h; (**ii**) isobutyric anhydride, 150 °C, 7 h; (**iii**) benzoyl chloride, 25 °C, 11 h.

### 2.2. Metabolic Stability

**Figure 2:**
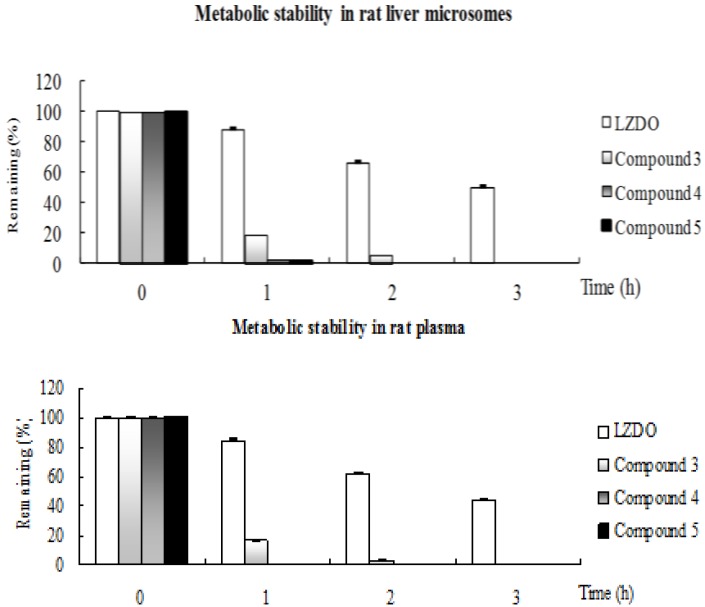
Metabolic stability of LZDO and prodrugs in rat liver microsomes and rat plasma.

Metabolic stability is an important property of drug candidate, since it affects parameters such as clearance, half-life, and bioavailability. A successful prodrug candidate is expected to undergo rapid, complete conversion to the parent compound in the plasma or microsomes within 1–3 h. Prodrugs **3**–**5** and parent LZDO (**1a**) were subjected to a metabolic stability study in the presence of rat liver microsomes and rat plasma. LZDO (**1a**) was fairly stable in rat liver microsomes and rat plasma, whereas prodrugs **3**–**5** were highly metabolized and converted to the desired parent compound **1a** in 1–3 h (Figure 2). Chemical degradation was not observed during metabolic stability study. The corresponding loss of prodrug compounds and formation of parent drug **1a** was determined by HPLC. 

Data are presented as the mean ± SD (n = 3). Percentage of prodrug remaining after metabolism was calculated by the ratio of the peak area at respective time (min) to peak area found at 0 min multiplied by 100: % remaining = [(peak area at respective time (min))/(peak area at 0 min)] × 100.

### 2.3. Formation of Parent Compound from Prodrugs in Rat Liver Microsomes and Rat Plasma

**Figure 3:**
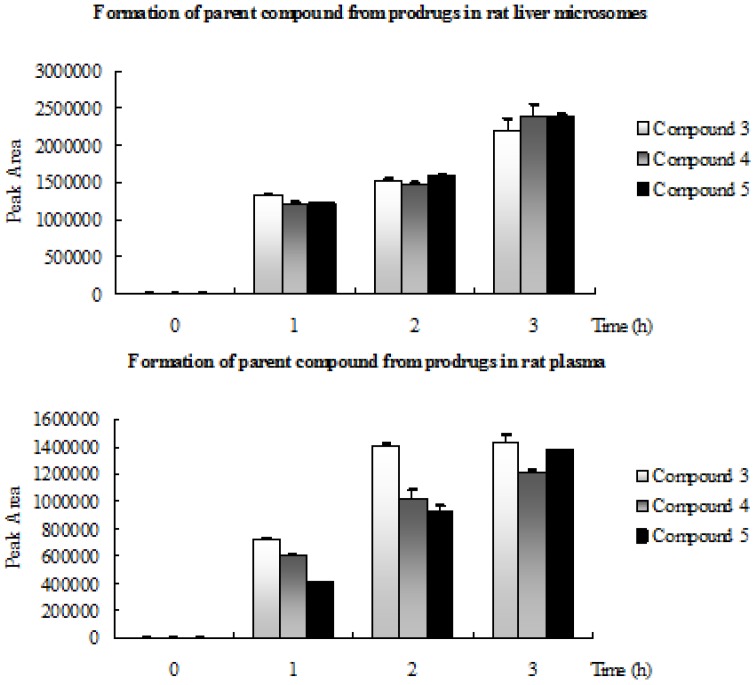
Formation of LZDO from prodrugs **3**–**5**. Data are presented as the mean ± SD (n = 3).

The experimental findings proved that all prodrugs were enzymatically labile and converted to parent compound LZDO as a metabolite by enzymatic hydrolysis of the ester group. The peak areas of the prodrugs in the HPLC analysis observed below detection level (BDL) after 3 h and correspondingly the parent drug LZDO peak area were increased (Figure 3).

### 2.4. Cardiac Function Screening

The cardiac contractility and safety of prodrugs **3**–**5** were evaluated on the myocardium in normal isolated rat hearts with LZDO as a positive control. Prodrugs **3**–**5** exerted positive inotropic effects in a dose-dependent manner. The increased rate in left ventricular developed pressure (LVDP, accepted as contractile force), +d*P*/d*t*_max_ (used as an index of contractility), the maximum rate of pressure decrease of the left ventricle (−d*P*/d*t*_min_, used as an index of diastolic function) and the heart rate (HR, used as an index of heart muscle oxygen consumption) of these compounds *vs.* control are listed in Table 1. 

As shown in Table 1, positive control LZDO and prodrugs **3**–**5** significantly increased LVDP, ±d*P*/d*t*_max_ at 1 *μ*M, 10 *μ*M, and 100 *μ*M, respectively (*p* < 0.05). The positive inotropic effect was reduced when LZDO was esterified. Prodrug **3** was the most potent prodrug for a positive inotropic effect on the myocardium. None of the compounds had a significant effect on HR (*p* > 0.05).

### 2.5. Application to a Pharmacokinetic Study

We selected prodrug **3** for a pharmacokinetic study because its positive inotropic effect *in vitro* was the highest among the three compounds. A thorough and complete method validation for determination of LZDO and prodrug **3** in rat plasma was validated for specificity, sensitivity, matrix effect, recovery, linearity, precision, accuracy and stability (data not shown).

**Table 1:** Effects of LZDO and prodrugs **3**–**5** on cardiac performance in isolated Langendorff-perfused rat hearts (mean ± SD, n = 6).

Compd	Group	LVDP	∆%	+d*P*/d*t*_max_	∆%	−d*P*/d*t*_max_	∆%	HR
	(*μ*M)	(mmHg)		(mmHg/s)		(mmHg/s)		(beat·min^−1^)
1a	0	50 ± 2.8		1,685 ± 147		−1,058 ± 81		183 ± 22
	1	60 ± 5 ^b^	19.4 ± 11.0	1,836 ± 121	9.4 ± 8.1	−1,175 ± 125	11.1 ± 10.0	186 ± 29
	10	73 ± 4 ^bd^	45.6 ± 9.0	2,125 ± 242 ^bc^	26.5 ± 13.4	−1,371 ± 186 ^bc^	30.3 ± 0.3	209 ± 21
	100	88 ± 3 ^bdf^	76.3 ± 12.3	2,702 ± 287 ^bdf^	60.9 ± 17.3	−1,911 ± 200 ^bdf^	82.0 ± 27.9	193 ± 15
3	0	55 ± 2.8		1,611 ± 27		−1,086 ± 38		207 ± 8
	1	63 ± 2 ^b^	15.0 ± 5.5	1,641 ± 29	1.9 ± 0.5	−1,139 ± 76	4.9 ± 5.9	203 ± 15
	10	69 ± 1.1 ^bd^	26.3 ± 7.7	1,768 ± 91 ^bd^	9.8 ± 7.0	−1,184 ± 75 ^a^	9.0 ± 5.1	204 ± 14
	100	79 ± 1.6 ^bdf^	44.7 ± 6.6	2,123 ± 82 ^bdf^	31.8 ± 5.3	−1,313 ± 70 ^bdf^	20.9 ± 5.1	205 ± 26
4	control	54 ± 5		1,634 ± 100		−1,044 ± 84		196 ± 21
	1	63 ± 3 ^b^	17.4 ± 9.8	1,669 ± 103	2.2 ± 3.4	−1,101 ± 102	5.6 ± 8.3	213 ± 46
	10	60 ± 4 ^a^	14.2 ± 10.0	1,765 ± 52 ^a^	4.9 ± 5.4	−1,196 ± 54 ^bc^	9.9 ± 9.5	196 ± 16
	100	73 ± 2.3 ^bdf^	20.2 ± 15.2	1,952 ± 49 ^bdf^	9.0 ± 8.9	−1,399 ± 61 ^bdf^	16.7± 15.2	191 ± 29
5	control	52 ± 4		1,671 ± 93		−1,081 ± 40		209 ± 47
	1	55 ± 5	5.3 ± 3.3	1,688 ± 95	1.0 ± 1.1	−1,095 ± 45	1.3 ± 0.8	189 ± 30
	10	59 ± 2.5 ^b^	13.9 ± 7.4	1,831 ± 14 ^bd^	9.9 ± 5.9	−1,245 ± 48 ^bd^	15.3 ± 2.8	196 ± 40
	100	64 ± 2.4 ^bde^	24.5 ± 11.2	1,962 ± 70 ^bdf^	17.6 ± 6.8	−1,314 ± 63 ^bde^	21.7 ± 7.2	213 ± 41

Each value is mean ± SD of six experiments. ^a^ *p* < 0.05, ^b^ *p* < 0.01 significantly different from control; ^c^ *p* < 0.05, ^d^ *p* < 0.01 significantly different from 1 *μ*M; ^e^ *p* < 0.05, ^f^ *p* < 0.01 significantly different from 10 *μ*M.

**Figure 4:**
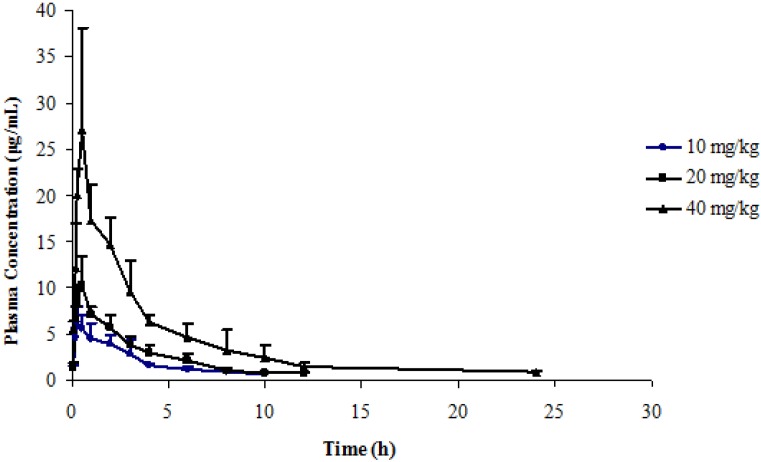
Time course of mean plasma LZDO concentrations (mean ± SD) after intragastric administering prodrug **3** at 10, 20 and 40 mg/kg in rats (n = 6).

Pharmacokinetic parameters including half-life, clearance, and bioavailability are important properties of a drug candidate. Figure 4 shows the mean plasma concentrations of LZDO after intragastric dosing at 10, 20 and 40 mg/kg of prodrug **3** in rats. The major pharmacokinetic parameters of LZDO were calculated using a noncompartment model based on statistical moment. The pharmacokinetic parameters of LZDO after intragastric administration of prodrug **3** at 10, 20 and 40 mg/kg in rats are summarized in Table 2. The pharmacokinetic parameters of LZDO after intragastric administering LZDO at 10, 20 and 50 mg/kg in rats are summarized in Table 3 [19]. Prodrug **3** was converted completely within 2 h in rats (data not shown).

As illustrated in the time-concentration and pharmacokinetic parameters profiles, prodrug **3** was highly metabolized and quickly converted to desired parent compound **1a**. Compared to LZDO, the T_max_ of prodrug **3** was higher than that of LZDO and the C_max_ of prodrug **3** was lower than that of LZDO, suggesting that prodrug **3** is converted into LZDO *in vivo*; the AUC of prodrug **3** after intragastric dose is comparable to that of LZDO, suggesting that the bioavailabilities of the prodrug **3** and LZDO were equivalent. Moreover, by comparison, the half-life (h) of LZDO increased to 4.01, 4.24, 4.39 for 10, 20, 40 mg/kg, respectively, and the clearance (L/h·kg) of LZDO was reduced to 0.40, 0.52, 0.41 for 10, 20, 40 mg/kg, respectively, suggesting that elimination of LZDO after taking prodrug **3** was relatively slower than after taking LZDO in rats, Therefore, absorption of LZDO after oral administration of the prodrug **3** is at least good as that of LZDO.

**Table 2:** Pharmacokinetic parameters of LZDO after intragastric administering prodrug **3** at 10, 20 and 40 mg/kg in rats (mean ± SD, n = 6).

Dose	10 mg/kg	20 mg/kg	40 mg/kg
T*_max_* (h)	0.42 ± 0.30	0.42 ± 0.13	0.42 ± 0.13
C*_max_* (mg/L)	6.50 ± 1.71	10.49 ± 3.14	28.39 ± 9.56
t_1/2_ (h)	4.01 ± 1.36	4.24 ± 1.52	4.39 ± 1.64
CL/F (L/h·kg)	0.40 ± 0.06	0.52 ± 0.08	0.41 ± 0.06
AUC_0–t_ (mg·h/L)	21.51 ± 2.82	33.90 ± 5.71	93.94 ± 13.76
AUC_0–∞_ (mg·h/L)	25.56 ± 3.82	38.86 ± 5.72	98.30 ± 15.37

**Table 3:** Pharmacokinetic parameters of LZDO after intragastric administering LZDO at 10, 20 and 40 mg/kg in rats (mean ± SD, n = 6) [19].

Dose	10 mg·kg^−1^ i.g.	20 mg·kg^−1^ i.g.	50 mg·kg^−1^ i.g.
T*_max_* (h)	0.29 ± 0.10	0.29 ± 0.10	0.29 ± 0.10
C*_max_* (mg/L)	8.58 ± 2.66	17.02 ± 5.50	49.33 ± 10.50
t_1/2_ (h)	2.21 ± 1.11	2.57 ± 2.14	1.78 ± 0.57
CL/F (L/h·kg)	0.79 ± 0.51	0.86 ± 0.57	0.98 ± 1.01
AUC_0–t_ (mg·h/L)	16.74 ± 8.90	34.84 ± 24.64	92.85 ± 64.10
AUC_0–∞_ (mg·h/L)	17.08 ± 8.99	35.93 ± 24.08	94.82 ± 66.88

## 3. Experimental

### 3.1. General

All commercial reagents and solvents were used without further purification unless otherwise specified. Melting points were determined using a model Buchi B-450 apparatus. ESI-MS were recorded on a TOF mass spectrometer (Applies-Biosystem Mariner). IR spectra were acquired by using a THERMO IR spectrometer Model Nicolet-100. ^1^H- and ^13^C-NMR spectra (500/125 MHz) were recorded on a Bruker Advance 500 spectrophotometer. Mass spectra were recorded on a ZAB-HS mass spectrometer and are reported as *m/z* values. HPLC analysis was conducted on a Waters 2695 HPLC instrument equipped with a UV detector (model 2489). LZDO and prodrugs **3**–**5** were analysed by HPLC, and their purity was confirmed to be greater than 98.0%. *In vitro* metabolic stability analysis was conducted by a HPLC method, using stock standard solutions of LZDO and prodrugs that were prepared in water at a concentration of 30 mM. The positive inotropic effects on the myocardium of LZDO and prodrugs **3**–**5** *in vitro* were evaluated on Langendorff-perfused hearts isolated from normal Sprague-Dawley rats. A HPLC method was applied to measure the rat plasma concentrations of LZDO and prodrug **3** after intragastric administration of prodrug **3** in the rats. 

### 3.2. Chemistry

*2,5-Di-iso-butyryloxymethyl-3,6-dimethylpyrazine* (**4**)*.* Compound **2** (16.8 g, 0.10 mol) was treated with isobutyric anhydride (200 mL, 0.32 mol) at 150 °C for 7 h. Reaction progress was monitored by TLC. After reaction completion, the excess propionic anhydride was evaporated. The residue was purified by silica gel column chromatography (petroleum ether-ethyl acetate = 4:1 v/v) to afford **4** as a yellowish oil (4.74 g, 15.4% yield, HPLC > 98%). ESI-MS (M+H^+^) calcd for C_16_H_25_N_2_O_4_ 309.1808, found 309.2153; UV max (methanol): 279 nm; IR (KBr) cm^−1^: 2978 (υ_CH_), 1716 (υ_C=O_), 1097 (ν_C-O_), 1461, 1417, 1296 (δ_CH_); ^1^H-NMR (CDCl_3_, 500 MHz) δ: 5.29 (s, 4H, 2×CH_2_-O), 2.71 (s, 6H, 2×CH_3_), 2.56 (q, 2H, *J* = 6.1, 2×CH-C=O), 1.05 (m, 12H, 2×CH(CH_3_)_2_) ppm; ^13^C-NMR (CDCl_3_, 125 MHz) δ: 173.0 (C=O), 149.8, 147.4 (C=N), 64.5 (C-O), 35.8 (C-C=O), 20.4 (CH_3_), 18.3 (CH_2_-CH_3_) ppm; EI-MS *m/z* (%): 308 (0.72, M), 237 (36.34, M-O=C-CH_2_-CH_2_-CH_3_), 167 (100, M-2×O=C-CH_2_-CH_2_-CH_3_+H).

*2,5-Dibenzoyloxymethyl-3,6-dimethylpyrazine* (**5**)*.* Benzoyl chloride (200 mL) was added dropwise into a solution of **2** (16.8 g, 0.10 mol) and the mixture was stirred at room temperature for 11 h. Reaction progress was monitored by TLC. After reaction completion, the reaction mixture was washed with saturated sodium bicarbonate solution (3 × 100 mL), extracted with ethyl acetate, dried over anhydrous sodium sulphate, filtered, and concentrated under vacuum. The residue was purified by silica gel column chromatography (petroleum ether-ethyl acetate = 3:1 v/v) affording **5** as white needle crystals (13.84 g, 36.8% yield, HPLC > 98%). mp 152–154 °C; ESI-MS (M+H^+^) calcd for C_22_H_21_N_2_O_4_ 377.1496, found 377.2237; UV max (methanol): 306 nm; IR (KBr) cm^−1^: 2947 (ν_CH_), 1728 (ν_O=C_), 1458 (δ_CH_), 1206 (ν_C-O_), 1102 (ν_C-C_), 886, 825 (δ_=CH_); ^1^H-NMR (CDCl_3_, 500 MHz) δ: 8.06 (d, 4H, *J* = 7.2 Hz, 2×Ph-H-2,6), 7.55 (t, 2H, *J* = 4.4 Hz, Ph-H-4), 7.44 (t, 4H, *J* = 7.2 Hz, 2×Ph-H-3,5), 5.26 (s, 4H, 2×CH_2_-O), 2.60(s, 6H, 2×CH_3_) ppm; ^13^C-NMR (CDCl_3_, 125 MHz) δ: 165.9 (C-O), 149.8, 147.4 (C=N), 133.1 (Ph-C-4), 131.0 (Ph-C-1), 129.9 (Ph-C-2,6), 128.8 (Ph-C-3,5), 64.2 (C-O), 19.8 (CH_3_) ppm; EI-MS *m/z*: 376 (M), 272 (M+H^+^-Ph-C=O), 168 (M+2H^+^-2×Ph-C=O), 105 (Ph-C=O+H^+^).

### 3.3. Metabolic Stability Analysis

Analysis was performed by using a Waters Alliance HPLC system (2695 pump, and 2489 PDA detector) with the following chromatographic parameters: wavelength, 278 nm; injection volume, 20 μL; run time, 20 min. The chromatographic separation was performed on an Phecda C_18_ (4.6 mm × 250 mm, 5 μm) column with a gradient elution of methanol and deionized water. Flow rate was 1.0 mL/min at 30 °C.

#### 3.3.1. Rat Plasma

Rat plasma was harvested from in-house rats. Fresh blood was collected from the male rat using a retro-orbital bleeding method in a tube containing heparin (100 IU/mL blood). After the collection of blood, plasma was separated from the blood by centrifugation at 5,000 rpm for 10 min. The supernatant plasma was separated and utilized for the further experiment.

##### *In Vitro* Physiological Stability of Prodrugs **3**–**5** and LZDO (**1a**) in Rat Plasma

The test compound in aqueous solution 30 mM (20 μL) was dissolved in rat plasma (980 μL). Immediately after addition (0 min), aliquots (100 μL) were removed and added to ice cold dissolvent (ethanol-ethyl acetate = 1:4 v/v, 1 mL) and mixed well by vortexing for 2 min. The mixture was centrifuged at 12,000 rpm for 10 min, and the supernatant was diluted with methanol and analyzed by HPLC. After 0 min, the remaining sample was incubated at 37 °C for 1, 2, and 3 h. After 1, 2, and 3 h, the sample (100 *μ*L) was treated with ice cold blending dissolvent (ethanol-ethyl acetate = 1:4 v/v, 1 mL), the mixture was vortex-mixed for 5 min, and then centrifuged at 5,000 rpm for 5 min. The upper organic layer (0.9 mL) was transferred to another tube and evaporated to dryness in a rotary evaporator (Centrivap console, Labconco Company, Kansas, MO, USA) at 40 °C. The supernatant was dissolved with 200 μL of water : methanol (30:70, *v/v*), vortex-mixed for 3 min, centrifuged at 12,000 rpm for 5 min, and a 20 μL of the supernatant was then injected into HPLC instrument.


% remaining = peak area at respective time (min)/peak area at 0 min × 100%
(1)

#### 3.3.2. Rat Liver Microsomes

Rat liver microsomes were prepared in house by a previously published method [23] and used immediately in the experiments. Protein concentrations were determined by a Lowry protein assay [24].

##### 3.3.2.1. Materials

Rat liver microsomes (2.0 mg/mL protein concentration), NADPH (1.0 mM solution, Sigma, Shanghai, China), Tris-HCl buffer (50 mM, pH 7.4, Sigma, Shanghai, China), test compound in aqueous solution (30 mM, 20 *μ*L). 

##### 3.3.2.2. Assay Procedure

Tris-HCl buffer (780 *μ*L), 1.0 mM NADPH solution (100 μL), and 100 μL of rat liver microsomes were mixed and vortexed at 37 °C for 5 min. To this mixture, 30 mM drug in aqueous solution (20 μL) was injected and vortexed well. Sample (100 μL) was immediately taken out (0 min) and transferred to the centrifuge tube containing ice cold dissolvent (ethanol-ethyl acetate = 1:4 v/v, 1 mL). The assay mixture was incubated in a water bath at 37 °C for 3 h and at specific time points (1 h, 2 h, 3 h). The assay mixture (100 μL) was taken out and added to the centrifuge tube containing an equal volume of cold dissolvent. Then all the tubes were vortexed and centrifuged at 12,000 rpm for 10 min. Aliquots of the supernatant were separated and used for analysis by HPLC. The percentage of prodrug remaining was calculated according to the following equation:

% remaining = peak area at respective time (min)/peak area at 0 min × 100%
(2)

### 3.4. *Ex Vivo* Langendorff-Perfused Rat Heart [25]

Sprague-Dawley adult male rats weighing 260–320 g were anaesthetised by intraperitoneal administration of 20% urethane (1.2 g/kg). The hearts were quickly removed and placed into an ice-cold modified buffer until contractions ceased. The heart was cleaned of surrounding tissues, mounted on the aortic cannula of the Langendorff perfusion system apparatus, and perfused with oxygenated buffer (mM): NaCl 117, KCl 5.7, CaCl_2_ 1.8, MgCl_2_ 1.7, NaHCO_3_ 4.4, NaH_2_PO_4_ 1.5, HEPES 20 and glucose 11. The buffer was equilibrated with 95% O_2_ and 5% CO_2_ and adjusted to pH 7.4 with NaOH before it entered the heart. An epicardial electrogram was registered using two stainless steel electrodes, one attached to the apex of the heart and the other to the aortic cannula. To obtain an isovolumetrically beating preparation, a latex balloon filled with water and connected by a catheter to a transducer was inserted through the left atrium into the left ventricle and inflated to provide an end-diastolic pressure between 8 and 12 mm Hg. Before each experimental protocol was initiated, the isolated hearts were set at a mean arterial pressure of 60–80 mm Hg and allowed to stabilise at 37 °C for 40–60 min. The isolated rat hearts were then perfused in sequence with 1, 10, and 100 μM of LZDO and prodrugs **3**–**5** in each group. A RM6240B/C four channel physiological recording instrument (Chengdu Instrument Factory, Chengdu, China) was used for continuous recording of LVSP, LVEDP, HR (derived from electrogram), +d*P*/d*t*_max_ and −d*P*/d*t*_max_ throughout the experiments. The criteria for established stability were LVDP > 50 mmHg, +d*P*/d*t*_max_ > 1,600 mmHg·s^−1^, heart rate > 180 beats·min^−1^ and normal sinus rhythm. The hearts were not studied if they had weak contractility (+d*P*/d*t*_max_ < 1,600 mmHg·s^−1^) and severe arrhythmia. Diastolic balloon pressure was maintained at 8 mmHg. LVDP was calculated as the difference between the systolic and diastolic pressures. The results are expressed as the mean value of hemodynamic variables (*n* = 6). Statistical analysis was performed using SPSS for Windows (version 13.0). Data were analyzed by One-way ANOVA. Values are given as mean ± SD and a p value less than 0.05 was considered as statistically significant.

### 3.5. Application to a Pharmacokinetic Study in Rats

Sprague-Dawley rats (male, weighing 200 ± 20 g) were obtained from Slack Laboratory Animal Site (Shanghai, China) and kept in an environmentally controlled breeding room for at least five days before experimentation. The rats were fasted overnight but with free access to water before the test. Animal welfare and experimental procedures were strictly in accordance with the guide for the care and use of laboratory animals [26]. After intragastric administration of prodrug **3** at 10, 20 and 40 mg/kg in rats, 100 μL of heparinized blood samples were collected at 0.08, 0.17, 0.25, 0.50, 1, 2, 3, 4, 6, 8, 10, 12 and 24 h from the ocular veins and immediately centrifuged at 5,000 rpm for 10 min. The supernatant plasma were collected and immediately frozen at −20 °C for not more than 7 days until analysis. The collected blood samples were pretreated by the aforementioned method (sample dilutions were made when necessary) and the concentrations of LZDO and prodrug **3** in each sample were finally determined based on the validated HPLC method. Pharmacokinetic parameters in rats were estimated by a noncompartmental method using DAS 2.0 software package (Mathematical Pharmacology Professional Committee of China, Shanghai, China).

## 4. Conclusions

The positive inotropic effect on the myocardium of LZDO and prodrugs **3**–**5** were evaluated by known experimental techniques. Prodrugs **3**–**5** exerted positive inotropic effects in normal isolated rat hearts, and prodrugs were transformed enzymatically to the parent drug **1a** in both rat liver chromosome and rat plasma. Pharmacokinetic studies showed that the half-life of the parent compound was extended to 4 h after intragastric administering prodrug **3** and absorption of LZDO after oral administration of the prodrug **3** wasat least good as for LZDO. On the basis of *in vitro* and *in vivo* studies, prodrug **3** was found to be a superior prodrug candidate for increasing myocardial contractility.

## Supplementary Materials

Supplementary materials can be accessed at: http://www.mdpi.com/1420-3049/18/4/4561/s1.
