# Supplementary material for: Synthesis, Biological Evaluation, and Pharmacokinetic Study of Novel Liguzinediol Prodrugs
Source: Molecules. 2013 Apr 18;18(4):4561–72. doi: 10.3390/molecules18044561 (PMC6270099; doi:10.3390/molecules18044561)

# Supplementary Materials

**Compound 4**

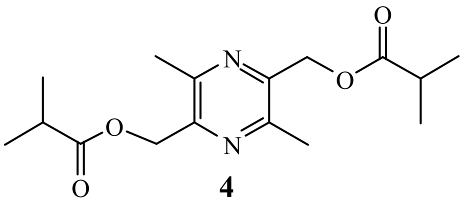


**4**

**Figure S1. Compound 4** $^{1}$H-NMR.

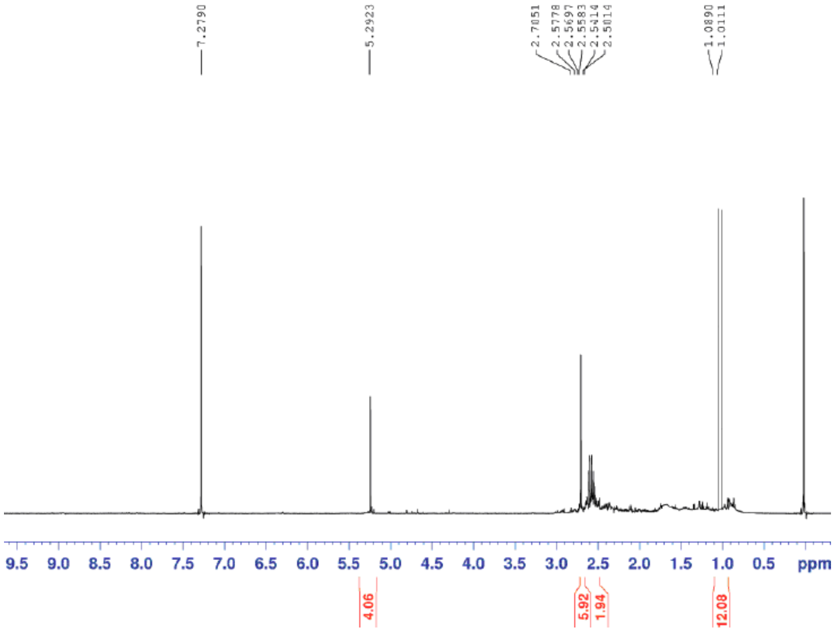


**Figure S2.** Compound **4** $^{13}$C-NMR.

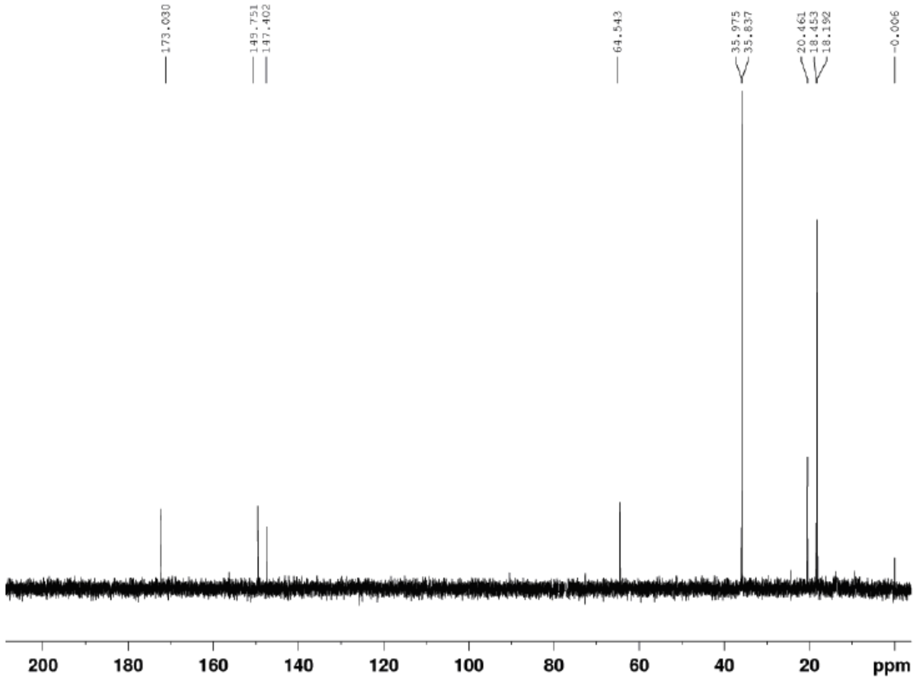

**Figure S3.** Compound **4** IR.

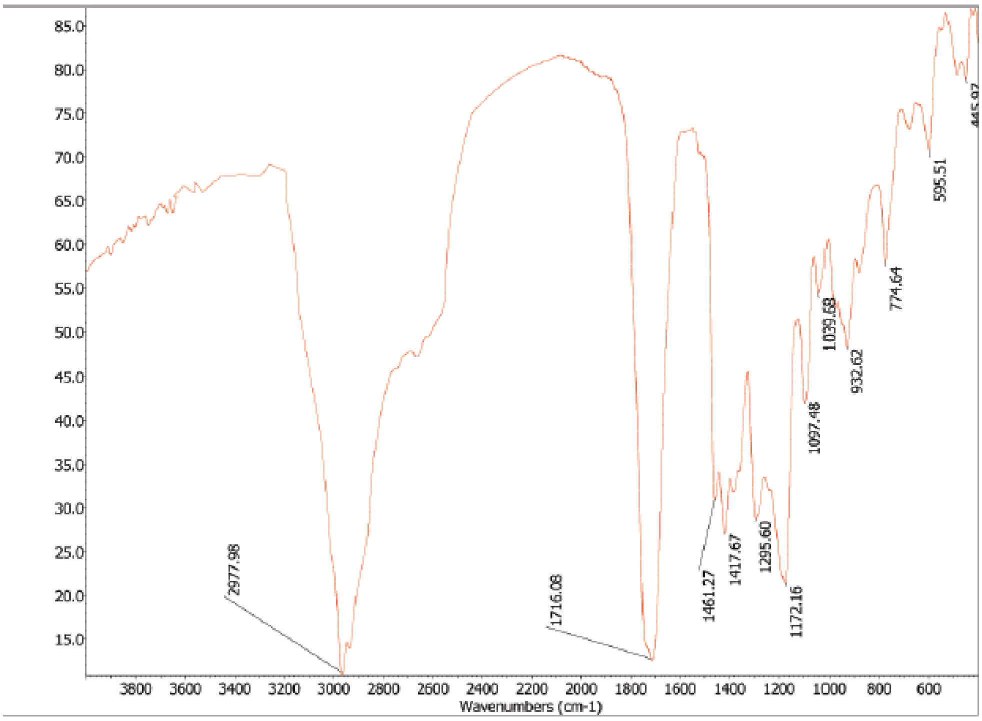


**Figure S4.** Compound **4** MS.

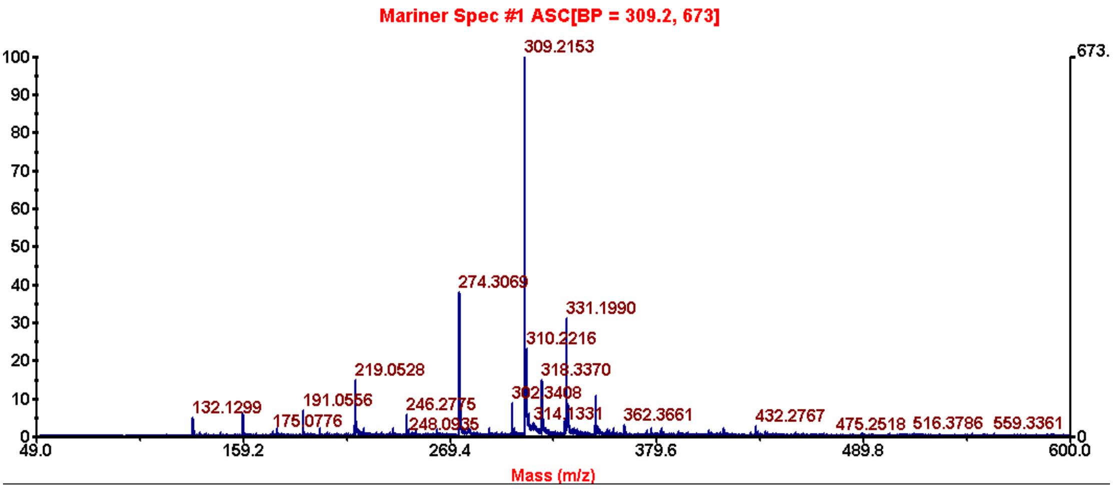

Compound **5**

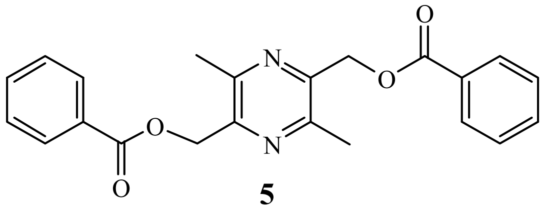


**5**

**Figure S5.** Compound **5** $^{1}$H-NMR.

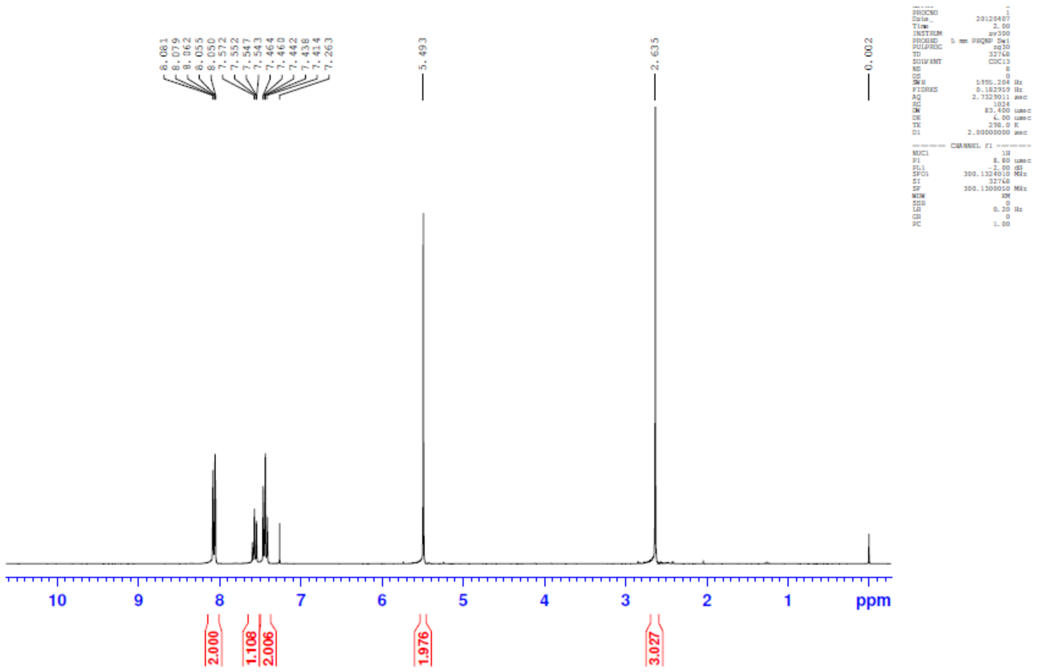


**Figure S6.** Compound **5** $^{13}$C-NMR.

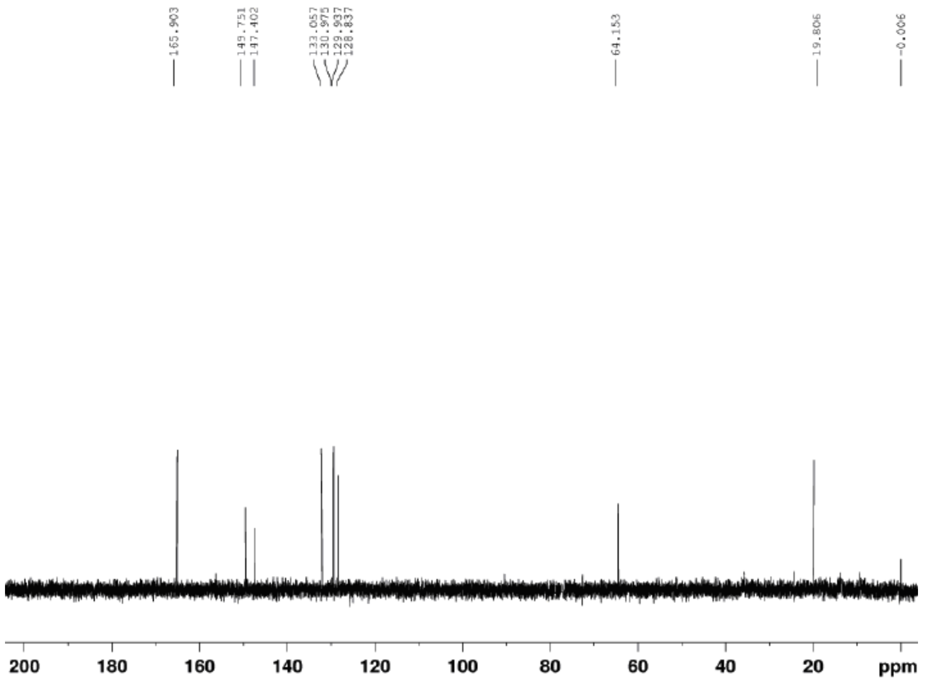

**Figure S7.** Compound **5** IR.

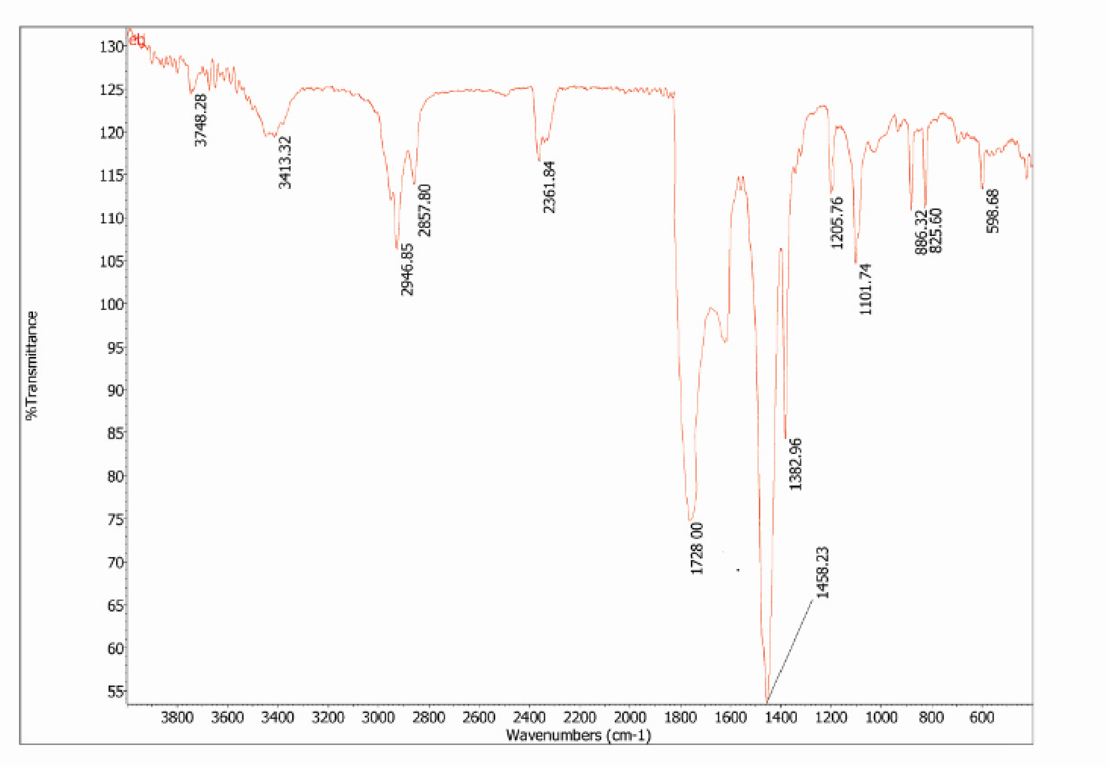


**Figure S8.** Compound **5** MS.

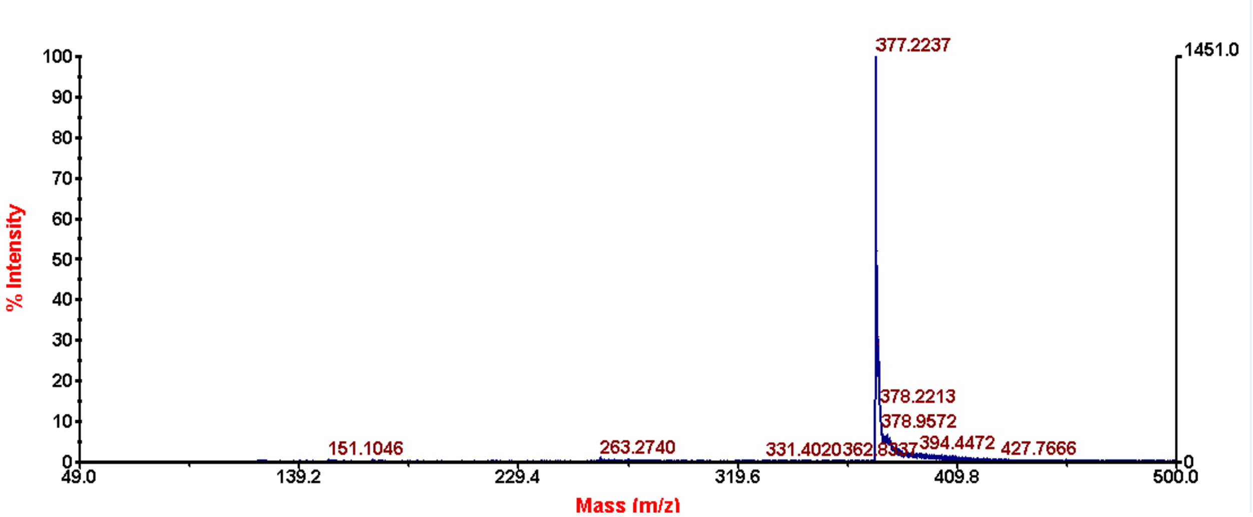

Supplement: Supplementary file 1 [file molecules-18-04561-s001.pdf]
